# Transcriptome analysis of *Pinus monticola* primary needles by RNA-seq provides novel insight into host resistance to *Cronartium ribicola*

**DOI:** 10.1186/1471-2164-14-884

**Published:** 2013-12-16

**Authors:** Jun-Jun Liu, Rona N Sturrock, Ross Benton

**Affiliations:** 1 Pacific Forestry Centre, Canadian Forest Service, Natural Resources Canada, 506 West Burnside Road, Victoria, BC V8Z 1 M5, Canada

**Keywords:** Defence response, Disease resistance, RNA-seq analysis, Transcriptome profiling, White pine-blister rust (WP-BR) interaction

## Abstract

**Background:**

Five-needle pines are important forest species that have been devastated by white pine blister rust (WPBR, caused by *Cronartium ribicola*) across North America. Currently little transcriptomic and genomic data are available to understand molecular interactions in the WPBR pathosystem.

**Results:**

We report here RNA-seq analysis results using Illumina deep sequencing of primary needles of western white pine (*Pinus monticola*) infected with WPBR. *De novo* gene assembly was used to generate the first *P. monticola* consensus transcriptome, which contained 39,439 unique transcripts with an average length of 1,303 bp and a total length of 51.4 Mb. About 23,000 *P. monticola* unigenes produced orthologous hits in the *Pinus* gene index (PGI) database (BLASTn with E values < e-100) and 6,300 genes were expressed actively (at RPKM ≥ 10) in the healthy tissues. Comparison of transcriptomes from WPBR-susceptible and -resistant genotypes revealed a total of 979 differentially expressed genes (DEGs) with a significant fold change > 1.5 during *P. monticola- C. ribicola* interactions. Three hundred and ten DEGs were regulated similarly in both susceptible and resistant seedlings and 275 DEGs showed regulatory differences between susceptible and resistant seedlings post infection by *C. ribicola*. The DEGs up-regulated in resistant seedlings included a set of putative signal receptor genes encoding disease resistance protein homologs, calcineurin B-like (CBL)-interacting protein kinases (CIPK), F-box family proteins (FBP), and abscisic acid (ABA) receptor; transcriptional factor (TF) genes of multiple families; genes homologous to apoptosis-inducing factor (AIF), flowering locus T-like protein (FT), and subtilisin-like protease. DEGs up-regulated in resistant seedlings also included a wide diversity of down-stream genes (encoding enzymes involved in different metabolic pathways, pathogenesis-related -PR proteins of multiple families, and anti-microbial proteins). A large proportion of the down-regulated DEGs were related to photosystems, the metabolic pathways of carbon fixation and flavonoid biosynthesis.

**Conclusions:**

The novel *P. monticola* transcriptome data provide a basis for future studies of genetic resistance in a non-model, coniferous species. Our global gene expression profiling presents a comprehensive view of transcriptomic regulation in the WPBR pathosystem and yields novel insights on molecular and biochemical mechanisms of disease resistance in conifers.

## Background

Genetic resistance to the white pine blister rust (WPBR) fungus (*Cronartium ribicola*) in western white pine (WWP, *Pinus monticola*) and other five-needle pines is an important and highly desired trait. Introduced to North America in the early 1900s, *C. ribicola* has decimated native white pines and significantly altered both forest ecosystems and the ability to manage the species for profitable timber production. White pine breeding and subsequent use of resistant germplasm for forest restoration is a long-term process; since the 1940s, it has required the attention of a few generations of forest geneticists [1].

Several types of DNA markers such as amplified fragment-length polymorphism (AFLP) markers [2], single nucleotide polymorphism (SNP) markers [3,4] and microsatellite (SSR) markers [4] have been developed and applied to WWP research, and there is some molecular information is available for molecular breeding of white pine resistance against *C. ribicola*[5]. For example, the plant disease resistance (R) family of NBS-LRR proteins and several families of pathogenesis-related (PR) proteins, including chitinases (PR3), thaumatin like proteins -TLPs (PR5), intracellular ribonuclease-like proteins (PR10), and anti-microbial peptides/proteins (AMPs), have been shown to contribute to host resistance in WP-BR interactions [5,6]. A recent proteomic profiling uncovered over one hundred *P. monticola* proteins modulated by *C. ribicola* inoculation, which included heat shock proteins (HSPs), reactive oxygen species (ROS) scavenging enzymes, and intermediate factors functioning in the signal transduction pathways triggered by well-known plant R genes, as well as other defence-related proteins [7]. Histochemical analysis revealed that the resistance response to systemic *C. ribicola* spread is localized internally in needle and stem tissues and that the build-up of physical barriers and deposition of cell wall-bound phenolic compounds play a crucial role in the defense reaction [8,9]. Despite these important results, there is still much to learn about the genetic basis of host resistance to *C. ribicola* in WWP and other five-needle pines (such as whitebark pine and limber pine).

Even though there have been significant improvements in genomic sequencing techniques over the past decade, the full genome of a conifer species is still unavailable. As a group, white pines have one of the largest plant genomes (27.36–37.68 pg/C) [10]; the genome size of *P. monticola* is estimated at 28.25 pg/C with a calculated length of about 2.7?×?10^4^ Mb per 1C genome. Full genome sequencing of any single white pine species would thus be very expensive. RNA sequencing (RNA-seq) is a recently developed, high-throughput method for profiling transcriptomes. RNA-seq is cost-economic and time-saving, especially compared to traditional expressed sequence tag (EST) sequencing, and it can generate transcriptome data for non-model species using incomplete genome information [11]. In addition to profiling gene expression, RNA-seq has shown powerful applications in areas, such as cataloguing of non-coding RNAs, investigation of the transcriptional structure of genes and splicing patterns, and the study of posttranscriptional modification and mutations [12]. RNA-seq has also provided information on complex regulation networks for gene expression patterns and on gene variations (such as SNPs and SSRs) in an increasing number of non-model plants [13], but, to date has not been used in study of the WPBR pathosystem.

In this study, we used RNA-seq analysis to profile the transcriptome of *P. monticola* primary needles during early stages of infection by *C. ribicola*; seedlings with major gene resistant (*Cr2/-*) and susceptible (*cr2/cr2*) genotypes were used. With *de novo* assembly followed by gene annotation and functional classification, our RNA-seq analysis generated the first *P. monticola* consensus transcriptome. Comparison of RNA-seq data sets from resistant (*Cr2/-*) and susceptible (*cr2/cr2*) genotypes revealed significant expressional differences among genes involved in defense signalling pathways and metabolic pathways. The first-ever set of transcriptome and global gene expression data reported here on *C. ribicola*-infected white pine needles significantly expands our knowledge of the molecular framework of the WPBR pathosystem.

## Results

### Transcriptome sequencing by RNA-seq and *de novo* assembly

Three RNA-seq 76-bp paired end read libraries were prepared from total RNA extracted from primary needles of uninfected seedlings (control at time 0-day post *C. ribicola*-infection, dpi) and infected seedlings with resistant (*Cr2/-*) and susceptible (*cr2/cr2*) genotypes (at 4-dpi). For each of three cDNA libraries, 116.4, 123.5 and 141.9 million 76-bp paired end reads were collected respectively. An overview of the raw reads data is given in Additional file 1: Table S1. Only 0.2% of raw reads were discarded due to low quality bases and reads (Additional file 1: Table S1). A separate assembly was performed for each of the three libraries and produced 39,135 to 45,236 contigs with an average N50 of 1,488-bp and an average length of 875-bp (Table 1). We also performed a single assembly of the total reads from all three cDNA libraries, which generated a common transcriptome data set of 36,923 contigs having a minimum length of 300-bp and a minimum mapped read of 50.

**Table 1:** **Measurement of contigs from *de novo *** **assembly**

**Sample ID**	**N75**	**N50**	**N25**	**Mini**	**Maxi**	**Ave**	**Count**	**Total (bp)**
**Assembly of three libraries separately**								
Un-infected	707	1,580	2,466	160	13,127	899	42,499	38,211,860
Res-Cr2/_ (4-dpi)	661	1,495	2,408	116	20,237	872	45,236	39,447,742
Sus-cr2/cr2 (4-dpi)	663	1,390	2,188	131	13,104	855	39,135	33,478,214
Average	677	1,488	2,354	136	15,489	875	42,290	37,045,939
**Consensus transcriptome**	**1,094**	**1,814**	**2,696**	**197**	**20,237**	**1,303**	**39,439**	**51,405,388**
**Assembly of three libraries together**								
	425	1113	2121	165	16,402	670	72,095	48,292,721
**Common transcriptome**^**(a)**^	**1712**	**2409**	**3376**	**300**	**16,402**	**1041**	**36,923**	**38,447,991**

^a)^contigs were selected with minimum length of 300-bp and minimum mapped reads of 50.

Since the separate assembly approach aligned more raw reads (75.5% of total) into contigs and mapped them back to the assembled transcripts than did the single assembly approach (Additional file 1: Table S1), a reciprocal BLASTn approach was used for search of one cDNA library assembly with another. This process generated a consensus transcriptome of 39,439 unique genes with each contig present in at least two cDNA libraries. This assembly of 39,439 contigs had an average length of 1,303-bp and a total length of ~51-Mb. The remaining contigs specific for each cDNA library were searched again by BLASTn against the PGI database, and those sample-specific contigs with strong Blastn hits (E-value?<?e-20) were added to the consensus transcriptome, producing a transcriptome of 43,890 contigs as reference for further analysis.

Using BLAST programs to assess the *de novo* assembly quality, the consensus transcriptome of 39,439 unique transcripts was compared with the PGI and Spruce Gene Index (SGI) databases, the protein database of the poplar leaf rust fungus *Melampsora laricis-populina,* as well as a set of *P. monticola* EST data (Additional file 1: Table S2). BLAST analysis revealed that 85% (~33,000 contigs) of infected *P. monticola* consensus assembly showed significant homology to the PGI and SGI databases (tBLASTx with E value?<?e-10), about 23,000 contigs (59% of the total) having orthologous hits in the PGI database alone. Only 2.8% of the infected WWP consensus transcriptome had orthologous hits in the poplar leaf rust fungus genome (BLASTx with E value?<?e-100), suggesting that ~5% of the assembled transcripts may come from *C. ribicola*. BLASTn analysis also revealed that ~97% of the *P. monticola* ESTs had orthologous hit in the *P. monticola* consensus assembly (E value?<?e-100) (Additional file 1: Table S2). These results indicate that we obtained a high percentage of *P. monticola* expressed genes and thus further support application of the *de novo* assembled transcriptome for global gene expression profiling.

### Transcriptional profile of western white pine primary needles

**Figure 1:**
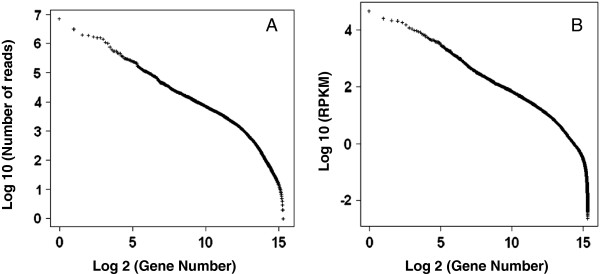
**Contig numbers and their expression values in the*Pinus monticola*transcriptome assembled from primary needles. (A)** Total gene reads were mapped to the genes. **(B)** RPKM values were mapped to the genes.

The top ten contigs with the highest expression values (Additional file 1: Table S3) of total gene reads accounted for 30% of total mapped reads and they were followed by about 4,600 contigs that were mapped by 90% of total mapped reads (Figure 1A). When gene expression levels were normalised by the reads per kilobase of transcript per million mapped reads (RPKM) values to get a more reasonable index for relative levels of transcript expression, the top 10 contigs with the highest RPKM values (Additional file 1: Table S3) were still mapped by 26% of total mapped reads. There were ~1,500 contigs with highly abundant expression levels at RPKM ≥50 and ~6,300 contigs with transcript levels at RPKM ≥10 in the control primary needles (un-infected at 0-dpi) (Figure 1B). The transcriptome set with 43,890 contigs appeared to be saturated as estimated by either total gene reads or RPKM. The top 10 unigenes with most abundant transcript expression were *P. monticola* homologs encoding for ribulose bisphosphate carboxylase/oxygenase (RuBisCO), rRNA intron encoded homing endonuclease, a chloroplastic oxygen-evolving enhancer protein, a chlorophyll a/b binding protein, S-adenosylmethionine-dependent methyltransferase, RuBisCO activase, fructose-bisphosphate aldolase, cell wall-associated hydrolase, as well as conserved hypothetical proteins with unknown function (Additional file 1: Table S3).

### Annotation of the primary needle transcriptome

**Figure 2:**
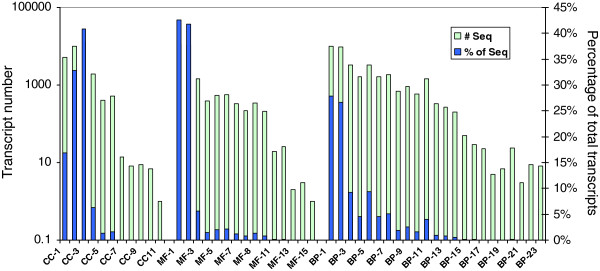
**Functional classification of the primary needle transcriptome of*Pinus monticola*assembled*de novo*from RNA-seq data based on gene ontology (GO).** Subcategories of cellular component (CC), molecular function (MF), and biological process (BP) are indicated as: CC-1, membrane; CC-2, organelle; CC-3, cell; CC-4, macromolecular complex; CC-5, extracellular region; CC-6, membrane-enclosed lumen; CC-7, extracellular matrix; CC-8, symplast; CC-9, cell junction; CC-10, virion; CC11, synapse; MF-1, binding; MF-2, catalytic activity; MF-3, transporter activity; MF4, electron carrier activity; MF-5, structural molecule activity; MF-6, nucleic acid binding transcription factor activity; MF-7, receptor activity; MF-8, antioxidant activity; MF-8, molecular transducer activity; MF-10, enzyme regulator activity; MF-11, nutrient reservoir activity; MF-12, protein binding transcription factor activity; MF-13, protein tag; MF-14, metallochaperone activity; MF-15, translation regulator activity; BP-1, metabolic process; BP-2, cellular process; BP-3, response to stimulus; BP-4, developmental process; BP-5, biological regulation; BP-6, multicellular organismal process; BP-7, localization; BP-8, signalling; BP-9, reproduction; BP-10, multi-organism process; BP-11, cellular component organization or biogenesis; BP-12, growth; BP-13, death; BP-14, immune system process; BP-15, cell proliferation; BP-16, rhythmic process; BP-17, biological adhesion; BP-18, carbon utilization; BP-19, nitrogen utilization; BP-20, locomotion; BP-21, cell killing; BP-22, pigmentation; and BP-23, viral reproduction.

Gene annotation was performed for the *P. monticola* primary needle transcriptome assembly based on sequence homologies to the databases of the National Center for Biotechnology Information (NCBI) non-redundant sequences (nr), the Protein Information Resource (PIR), the Universal Protein Resource (UniProts), the Gene Ontology (GO), and the Kyoto Encyclopedia of Genes and Genomes (KEGG) using BLAST2GO. Gene names and GO terms were assigned to the contigs based on their homologies to these available databases. About 72% of total contigs (31,577 out of 43,890) were assigned gene names and 50% of them (21,577) were assigned at least one GO term. GO annotation assignments classified 21,577 unique contigs into 23 subcategories of the biological process category, 15 subcategories of the molecular function category, and 11 subcategories of the cellular component category at level 2 (Figure 2). The four subcategories with the most highly abundant transcripts under the biological process category were metabolic process (27.8%), cellular process (26.6%), biological regulation (9.34%), and response to stimulus (9.2%). The subcategories with the most highly abundant transcripts in the molecular function category included binding (42.6%), catalytic activity (41.7%), and transporter activity (5.5%). Within the cellular components category, the four most common groups of proteins were assigned to the subcategories of cell (40.8%), organelle (32.8%), membrane (16.9%), and macromolecular complex (6.2%) (Figure 2).

**Figure 3:**
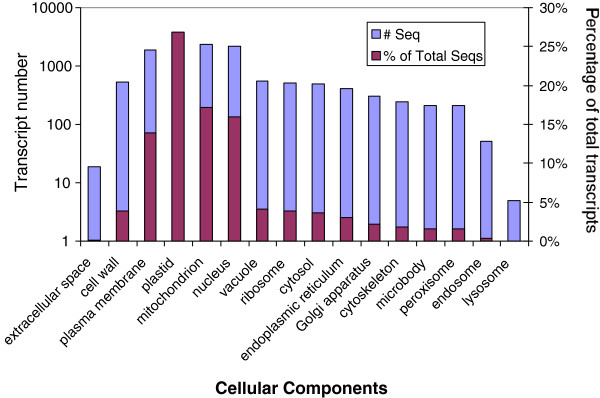
**Distribution of unique transcripts in the primary needle transcriptome of*Pinus monticola*assigned to GO cellular component category using “goslim_plant” analysis in the BLAST2GO program.** Sequences were assigned a putative function based on sequence identity to annotated proteins from other species.

To further estimate the completeness of the transcriptome data and the effectiveness of annotations, we searched annotated contig sequences using GOslim_plant. More than one quarter (26.9%) of the sequences were localized to the plastid, 17.1% to the mitochondrion, 15.9% to the nucleus, and 13.8% to the plasma membrane. The extracellular space and cell wall were localized by about 4% of total sequences, contributing to the first layer of plant defence to outside stimuli (Figure 3).

Gene annotation conducted using enzyme code and KEGG databases revealed activities of many biological pathways in *P. monticola* primary needles. A total of 1,315 enzymes encoded by 7,561 transcripts were mapped to 136 metabolic pathways (Additional file 1: Table S4). Six pathways with the most abundant unique sequences included starch and sucrose metabolism (5.9%), purine metabolism (5.8%), phenylalanine metabolism (3.4%), methane metabolism (3.4%), phenylpropanoid biosynthesis (3.2%), and amino sugar and nucleotide sugar metabolism (2.6%). Each of these metabolic pathways was mapped with at least 200 unique transcripts.

### Detection of differentially expressed genes (DEGs) in response to rust infection

**Figure 4:**
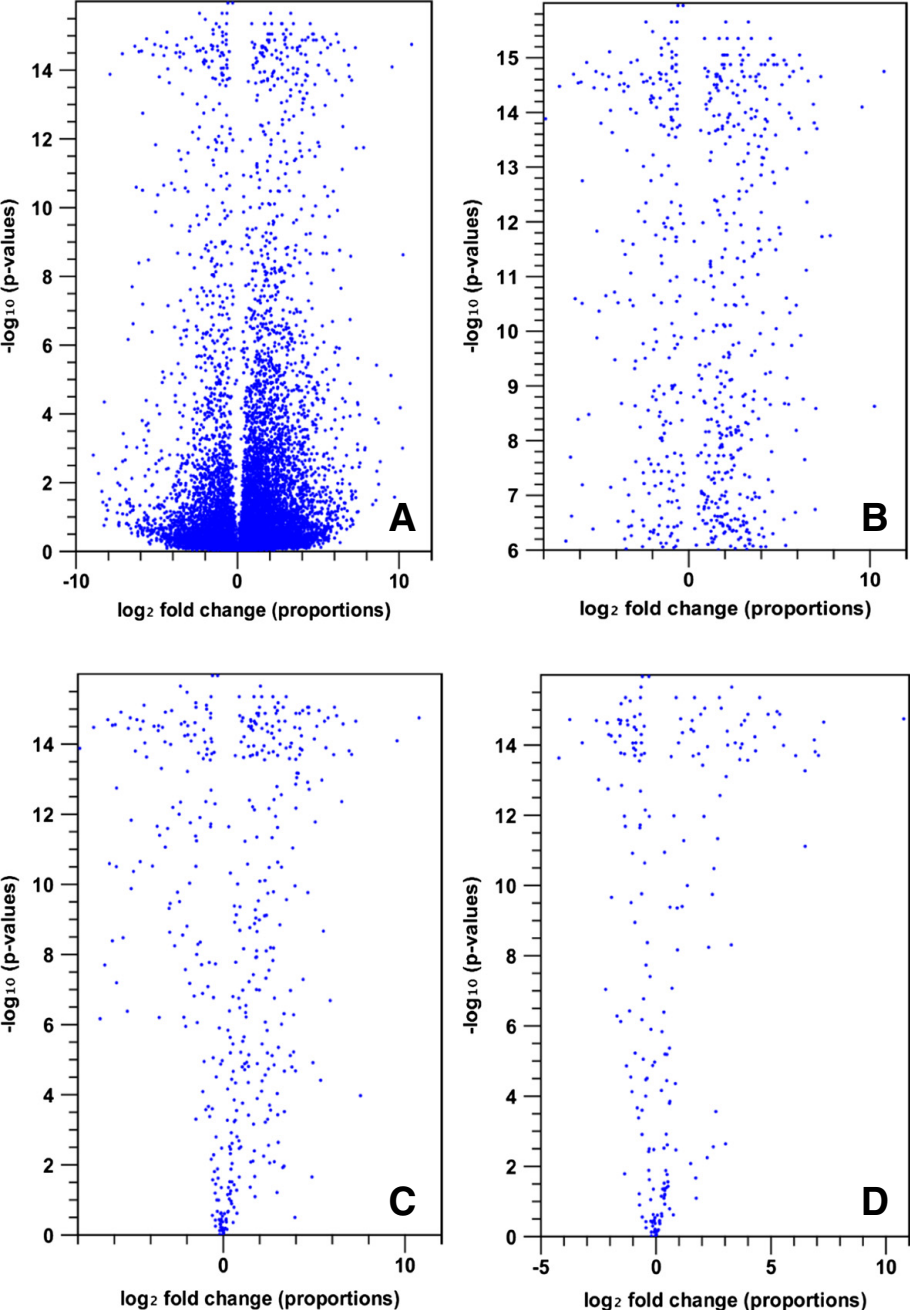
**Global view of transcriptional changes by volcano plot using Kal’s statistical test (Z-test).** Log2 fold change in RPKM expression values (x-axis) versus -log10 (Bonferroni corrected P-values) (y-axis) were computed in scatter plots: **(A)** All 43,980 contigs in comparisons of three cDNA libraries; **(B)** Differentially expressed genes (DEGs) detected between un-infected (0-dpi) and resistant seedlings post rust infection (4-dpi); **(C)** DEGs detected between un-infected (0-dpi) and susceptible seedlings post rust infection (4-dpi); and **(D)** DEGs detected between resistant and susceptible seedlings post rust infection.

**Figure 5:**
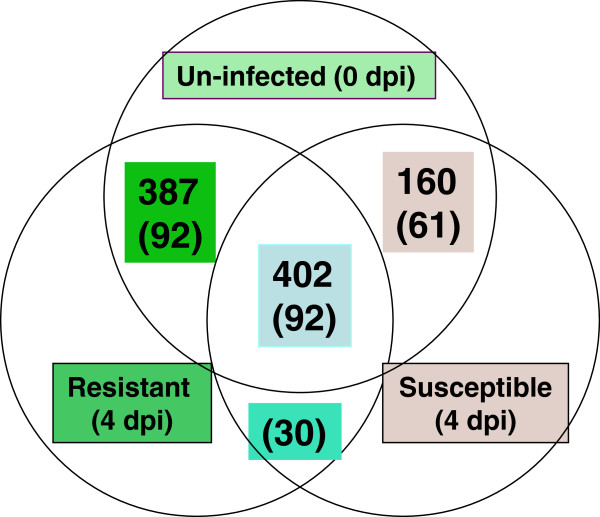
**Venn diagrams of differentially expressed genes (DEGs) in white pine-blister rust (WP-BR) interaction for illustrating the relationship of DEGs in two western white pine genotypes (*Cr2/-*and*cr2/cr2*) post rust infection.** The numbers of DEGs detected between resistant (*Cr2/-*) and susceptible (*cr2/cr2*) seedlings are shown in parentheses.

A quality control test on the data assembled from each cDNA library confirmed that they were suitable for statistical analysis for DEG identification (Additional file 2: Figure S1). We compared three WWP primary needle transcriptome profiles (*Cr2*-resistant seedlings at 4-dpi vs. control at 0-dpi, *cr2*-susceptible seedlings at 4-dpi vs. control at 0-dpi, and *Cr2*-resistant seedlings at 4-dpi vs. *cr2*-susceptible seedlings at 4-dpi) to better understand the WPBR pathosystem at the transcriptome level. The reference transcriptome with 43,890 contigs was used to map raw reads for DEG detection between any two treatments. A total of 979 DEGs were revealed with a RPKM fold change?>?1.5 and a cut-off of *p*?<?0.05 with Z-test by Bonferroni-correction (Figure 4). We detected 562 DEGs in compatible WP-BR interaction (*cr2 vs. avcr2*) and 789 DEGs in incompatible WP-BR interaction (*Cr2 vs. avcr2*) (Figure 5). There were 310 DEGs regulated similarly after *C. ribicola* infection in both susceptible and resistant seedlings while there were 275 DEGs regulated differently (Figures 4 and 5).

**Figure 6:**
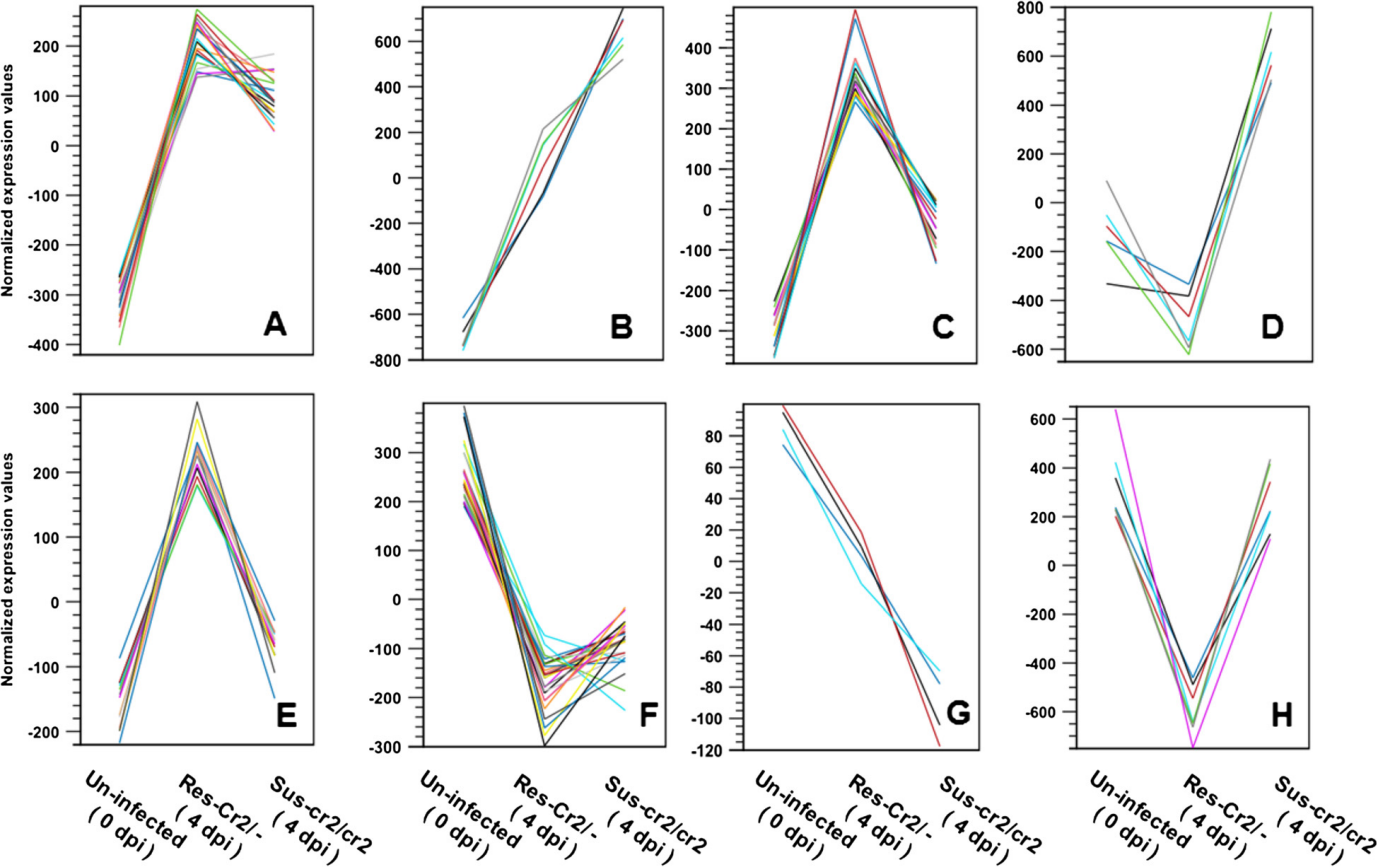
**Representatives of expression patterns of the differentially expressed genes (DEGs) by K-means clustering analysis in white pine-blister rust (WP-BR) interactions.** Genes used the clustering analysis are shown in the (Additional file 5: Table S14). **(A)** DEGs up-regulated similarly in both resistant and susceptible seedlings; **(B)** DEGs up-regulated higher in susceptible seedlings than in resistant seedlings; **(C)** DEGs up-regulated higher in resistant seedlings than in susceptible seedlings; **(D)** DEGs up-regulated only in susceptible seedlings; **(E)** DEGs up-regulated only in resistant seedlings; **(F)** DEGs down-regulated similarly in both resistant and susceptible seedlings; **(G)** DEGs down-regulated more in susceptible than in resistant seedlings; **(H)** DEGs down-regulated only in resistant seedlings.

The expression patterns were clustered into eight different types based on the K-means method: five types for up-regulation patterns (Figure 6A-E) and three types for down-regulation patterns (Figure 6?F-H). The type I cluster included DEGs regulated positively in both resistant and susceptible seedlings at similar magnitudes (Figure 6A). While types II and III DEGs also showed up-regulation in both resistant and susceptible seedlings, they differed in degree of up-regulation (Figure 6B and C). The type IV cluster included DEGs with rust-enhanced transcript levels only in susceptible seedlings (Figure 6D) and type V included DEGs enhanced only in resistant seedlings (Figure 6E). Down-regulated patterns after *C. ribicola* infection are represented by types VI - VIII. DEGs down-regulated at similar levels in both resistant and susceptible seedlings were grouped into the type VI (Figure 6F). The type VII included DEGs with greater down-regulation levels in susceptible than in resistant seedlings (Figure 6G). The type VIII included DEGs regulated negatively by rust infection only in resistant seedlings (Figure 6H).

**Figure 7:**
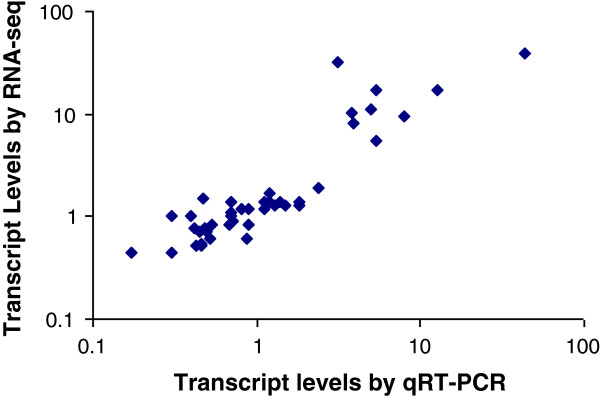
**Scatter-plot comparison of the fold changes of transcript levels measured by RNA-seq analysis (y axis) and quantitative reverse-transcriptase-polymerase chain reaction (qRT-PCR) (x axis).** Correlation was based on transcript expression regulated by *Cronartium ribicola* (4 days post infection vs. uninfected control seedlings) for 26 genes in resistant (*Cr2/-*) and susceptible (*cr2/cr2*) genotypes respectively. Genes used the qRT-PCR analysis are shown in the (Additional file 5: Table S15).

To confirm gene expression level measured by RPKM fold change, a subset of 26 contigs were subjected to analysis of quantitative reverse-transcriptase polymerase chain reaction (qRT-PCR). As shown in Figure 7, the relative transcript levels measured by qRT-PCR and RNA-seq were highly correlated (R?=?0.8) with statistical significance (*p*?=?0).

To explore potential functions of DEGs in response to *C. ribicola* invasion, GO-based classification was conducted. Eighty-five percent (830/979) of all DEGs showed significant homologies with expressed genes from other organisms. GO analysis classified 508 of the annotated DEGs into 17, 13, and 8 subgroups (at level-2) of the biological process, molecular function, and cellular component categories, respectively (Additional file 3: Figure S2). The three biological processes most affected by *C. ribicola* infection were in the subcategories of metabolic process (345 DEGs), cellular process (303 DEGs) and response to stimulus (174 DEGs). Among the 136 metabolic pathways identified as active in infected white pine needles, 90 of them were affected significantly by rust infection. Only ~5.4% of total annotated contigs (410/7,561) were regulated by rust infection, but they encoded ~20.5% of total annotated enzymes (270/1,315) in the primary needle transcriptome. Based on their high sequence and enzyme numbers, the top 10 rust-modulated pathways were flavonoid biosynthesis, methane metabolism, carbon fixation in photosynthesis, starch and sucrose metabolism, phenylalanine metabolism, phenylpropanoid biosynthesis, amino sugar and nucleotide sugar metabolism, glycolysis/gluconeogenesis, cysteine and methionine metabolism, and pyruvate metabolism (Additional file 1: Table S4).

### DEGs specifically regulated in resistant seedlings

A total of 789 DEGs were detected in the comparison of the resistant genotype (*Cr2/-*) at 4-dpi with control at 0-dpi. Of these 789 DEGs, 387 were regulated only in resistant seedlings while another 402 genes were also regulated by rust infection in susceptible (*cr2/cr2*) seedlings (Figure 5). Of the 387 resistance-specific DEGs, 245 were up-regulated with fold changes up to 1,217 and one transcript by *de novo* synthesis (Additional file 4: Table S5) and 142 were down-regulated with fold changes down to −88 by rust infection (Additional file 4: Table S6). The up-regulated DEGs included a set of putative signal receptor genes encoding putative R proteins with domains of nucleotide-binding site (NBS) and leucine-rich repeat (LRR), receptor-like protein kinases (RLK), calcineurin B-like (CBL)-interacting protein kinases (CIPK), F-box family proteins (FBP), and abscisic acid (ABA) receptor; transcriptional factor (TF) genes of multiple families (such as NAC, DOF, PLATZ, ARF2, zinc-finger HD, nuclear transcription factor Y, and other DNA-binding proteins); genes homologous to apoptosis-inducing factor (AIF), flowering locus T-like protein (FT), and subtilisin-like protease; as well as a wide diversity of down-stream genes (encoding enzymes involving different metabolic pathways, PR proteins of multiple families, and AMPs) (Additional file 4: Table S5). A large proportion of down-regulated DEGs were related to photosystem I and II, the metabolic pathway of carbon fixation, and flavonoid biosynthesis (Additional file 1: Table S4 and Additional file 4: Table S6). Expansins are an example of cell-wall proteins down-regulated by rust infection in resistant seedlings.

### DEGs specifically regulated in susceptible seedlings

A total of 562 DEGs modulated by *C. ribicola* infection (at 4-dpi) were detected in the comparison of the susceptible (*cr2/cr2*) genotype with un-infected control seedlings. One hundred sixty of these DEGs were specifically regulated in susceptible seedlings, 114 genes of them up-regulated with fold changes up to 336 (Additional file 4: Table S7) and 46 genes down-regulated with fold changes down to −15 (Additional file 4: Table S8). In additional to a TF gene with a WRKY domain, we detected up-regulated genes for subsets of calcium-binding proteins, chitinases (PR3), TLPs (PR5), other PR proteins (PR1, PR4, and PR6), and myrosinase-binding proteins (MyroBP) specifically in susceptible seedlings (Additional file 4: Table S7). Apart from genes involved in photosystems, transcripts from multiple types of retrotransposons were noted among the down-regulated genes in the susceptible seedlings (Additional file 4: Table S8).

### DEGs regulated similarly in both resistant and susceptible seedlings

Of the 402 DEGs co-modulated in both resistant and susceptible genotypes, 310 of them showed no significant difference between both genotypes; 204 of these DEGs were up-regulated and 106 DEGs down-regulated following rust infection (Additional file 4: Table S9 and S10). A number of zinc-finger, RING-finger, FBP genes, and TFs (C3HL, AP2-REF-B3, R2R3-MYB, CCCH type, and HD-leucine zipper) were up-regulated in both genotypes, as well as transcripts encoding for subsets of proteins regulated in auxin-mediated signalling, dnaj chaperone family protein, glutathione S-transferases (GST), peroxidises, thioredoxins, chtinases, ubiquitin-conjugating enzyme e2, β-glucanases, glucanase-inhibitors, and enzymes related to biosyntheses of ethylene, phenylpropanoid, and stilbenoid (Additional file 4: Table S9), suggesting their involvement in basal host response to *C. ribicola* invasion. In addition to transcripts for a group of small heat-shock proteins (HSP), genes for photoassimilate-responsive proteins and enzymes related to carbon fixation in photosynthesis and flavonoid biosysthesis were among the 106 DEGs negatively regulated in both resistant and susceptible seedlings (Additional file 1: Table S4 and Additional file 4: Table S10).

### DEGs regulated differentially in both resistant and susceptible seedlings

A total of 275 DEGs were detected in the comparison between resistant and susceptible genotypes; 141 DEGs showed relatively higher transcript levels in resistant seedlings than in susceptible seedlings (Additional file 4: Table S11) while 134 DEGs showed the opposite expression pattern (Additional file 4: Table S12). Although carbon fixation in photosynthesis is down-regulated in both resistant and susceptible seedlings, the relative expression levels of related transcripts were higher in resistant seedlings, suggesting that rust infection caused less damage to photosynthesis in resistant seedlings than in susceptible seedlings. Relatively higher levels of transcripts related to biosynthesis of cellulose, flavonoid, flavone and flavonol in resistant seedlings suggested that more active production of these compounds may be beneficial to trees infected by WPBR. In contrast, relatively lower levels of transcripts in resistant than in susceptible seedlings indicated that compatible WP-BR interactions may lead to more active metabolisms for phenylalanine, tyrosine, starch and sucrose (Additional file 1: Table S4).

## Discussion

We used RNA-seq technology to generate transcriptome data and examined global gene expression profiles to identify defense-responsive genes in WP-BR interactions. This work demonstrates that RNA-seq is a useful and effective tool for *de novo* transcriptome assembly and discovery of candidate genes underlying host genetic resistance to pathogens, even in a non-model species without genome and complete EST databases. Enzyme annotation and pathway assignment of the *P. monticola* transcriptome provides a genomics resource for further investigating candidate genes involved in various metabolic pathways in a conifer species, such as those involved in physiological responses to environmental stresses. Comparison of sequence data from infected vs. non-infected and resistant vs. susceptible transcriptomes revealed almost one thousand DEGs using Z-test with a Bonferroni correction (2.5% of the total transcriptome assembly), and 85% of them were functionally annotated. It is noteworthy that a number of the DEGs revealed by RNA-seq include *P. monticola* defence-related genes/proteins investigated in previous studies, such as NBS-LRR, PR3, TLP (PR5), PR10, AMP, HSP, and other defence-related genes [5,7,14]. qRT-PCR analysis of a subset of DEGs further verified transcript expression levels as revealed by transcriptome comparison from raw RNA-seq data. These consistent results from various research strategies indicate that RNA-seq provides a powerful tool for comprehensive transcriptome profiling that reveals important molecular interactions in the WPBR pathosystem.

### *Cr2* candidates for incompatible WP-BR interaction

Plants have evolved and adapted various defense mechanisms to protect themselves from invasions by microbial pathogens. Plant immunity is controlled by two layers of inducible responses: basal response triggered by conserved microbial features, and specific response triggered by gene-for-gene recognition. Specific responses to pathogen attack activate effector-triggered immunity (ETI) through direct or indirect interaction of host R proteins with pathogen effector/ avirulence proteins, which leads to the hypersensitive response (HR), a defense mechanism featured by programmed cell death (PCD) around the local pathogen infection sites [15]. White pine *Cr2*-seedlings display a typical HR in response to infection by *C. ribicola avcr2* strain, presumably by recognizing the *avcr2* product using an uncharacterized receptor encoded by the *P. monoticola* R gene *Cr2*[16]. In other well-characterized pathosystems, the incompatible interaction usually leads to a series of dramatic molecular changes such as ion flux, a ROS burst, callose deposition, and eventually HR-like cell death, resulting in complete resistance to avirulent pathogenic strains [17]. In contrast, susceptible plants show only basal responses through interaction of host pattern recognition receptors (PRRs) with pathogen- or microbe-associated molecular patterns (PAMPs or MAMPs). In PAMP- or MAMP-triggered immunity (PTI), pathogen avirulence factors (Avr) promote virulence by targeting other host proteins (in the absence of R proteins) to manipulate plant physiological processes more suitable to pathogen growth and multiplication in susceptible plants [15].

Deciphering the transcript fingerprint of *Cr2* is one of the key questions in studying the molecular WP-BR interactions. In this study, we identified two NBS-LRR genes (with the highest identities to *Picea sitchensis* proteins ABR16233 and ABR16103, respectively) and two RLK genes (one RLK with extracellular LRR domain and another one with cysteine-rich domain) with significant up-regulation only in resistant seedlings post *C. ribicola* infection (Additional file 4: Table S5), which provides novel insight into the expression profiles of these two plant superfamilies in the WPBR pathosystem [6,7,18]. Most characterized plant R genes belong to the NBS-LRR or RLK superfamily for plant R-Avr interactions [19,20]. Up-regulation of RLK and NBS-LRR homologous transcripts in resistant seedlings by infection of the avcr2 rust strain suggests their involvement in white pine major gene (*Cr2*) resistance. SNP genotyping of both *P. monticola* NBS-LRR and RLK superfamilies for positional R candidates would help resolve the molecular characterization of *Cr2*.

Downstream signalling networks triggered by incompatible R-Avr interactions are mediated by calcium-activated protein kinases (such as CIPKs) and mitogen-activated PK (MAPK) cascades [21]. These signalling networks then control the activities and synthesis of a series of TFs, enzymes, phytohormones (such as jasmonic acid -JA and salicylic acid -SA), PR proteins, AMPs, phytoalexins and other secondary metabolites, which coordinately contribute to host resistance against pathogens [22]. Consistent with this widely accepted R-Avr interaction model, we detected significant up-regulation of transcripts for MAPK, CIPK, SNF1-related PK regulatory subunit, and other PKs in *P. monticola* resistant seedlings following rust infection. CBLs act as Ca2+ sensors to activate specific PKs. Rice CIPKs participate in various layers of MAMP-induced defense responses, leading to final PCD in rice [23]. Arabidopsis MAPKs (MPK3 and MPK6) are positive mediators of defense responses induced by MAMP and pathogen, controlling both basal resistance and elicitor-induced resistance to fungal pathogen *Botrytis cinerea*[24]. As a regulatory subunit of the SNF1-related protein kinase (SnRK) complex, SNF1-related protein kinase regulatory subunit beta-2 likely plays a role in a signal transduction cascade regulating gene expression and carbohydrate metabolism in plant response to environmental stresses. SnRK2s phosphorylate Ser/Thr residues in the R-X-X-S/T motif of the ABF type TFs for activation of a large number of ABA/stress-responsive genes [25]. Rust-upregulated transcript expression suggests that several *P. monticola* PK families, including MAPKs, CIPKs and SnRK2s (Additional file 5: Table S13), may be intermediate factors involved in *Cr2*-mediated resistance to *C. ribiciola*.

### Novel *P. monticola* genes involved in the *Cr2*-mediated resistance

Among DEGs positively regulated in *Cr2*-mediated resistance, *P. monticola* genes homologous to genes encoding AIF, FT-like protein, subtilisin-like protease, RING/FYVE/PHD zinc finger-containing proteins, and membrane attack complex component/perforin (MACPF) domain proteins, attracted our attention (Additional file 5: Table S13). The MACPF domain proteins are well-known for their critical functions in innate and adaptive immunity, and they are capable of forming transmembrane lytic pores known as the membrane-attack complex (MAC) by interacting with other cell components for pathogen invasion or host protection. Arabidopsis MACPF proteins probably act as mediators that recognize plant signals for negative regulation of cell death programs and defense responses [26,27]. Both SA and a chitin elicitor promote expression of an Arabidopsis MACPF gene (*CAD1*) and the *cad1* mutant shows spontaneously activated expression of PR genes and greatly increased SA levels [26]. RING finger proteins constitute a large family and play key roles in regulating plant growth/developmental processes, hormone signalling, and responses to (a)biotic stresses. A rice RING-H2 finger gene (*OsBIRF1*) was induced differentially in an incompatible interaction with *Magnaporthe grisea*, and its constitutive expression led to enhanced disease resistance and elevated expression levels of defense-related genes encoding PR1, PR2, PR3 and PR5 proteins [28]. Up-regulation of a *P. monticola* MACPF homolog and differential expression of the *P. monticola* RING gene family in the incompatible WP-BR interaction suggest their potential roles in conifer defense against *C. ribicola*.

As a positive regulator of apoptosis in mammals, AIFs function in apoptotic and redox signalling: which enhances mitochondrial bioenergetics and complex I activity/assembly to help maintain proper cellular redox homeostasis in mitochondria and forms a chromatin degrading complex with other proteins in the nucleus [29]. In addition to up-regulation of an AIF homolog in the incompatible WP-BR interaction, we also observed differential expression of families of thioredoxins, GSTs and peroxidises (Additional file 5: Table S13), suggesting that redox signalling by oxidative burst is part of the defence mechanism of *P. monticola* against *C. ribicola*[8]. Recently a unique peptide signal (GmSubPep), embedded in a soybean subtilisin-like protein, was discovered to activate the transcription of defense genes against pathogens [30]. Although transcript levels of two subtilisin-like protein homologs were significantly up-regulated in *Cr2*-mediated resistance (14- and 80-fold), whether these proteins are processed to generate signal peptides involved in host defence is still an interesting question to address in future studies. Plant FT RNA is able to travel by its *cis*-element from leaf to shoot apical meristem [31], suggesting its role in systemic signalling by intercellular RNA trafficking through phloem transport. RNA trafficking contributes to local and long-distance coordination of plant development and response to the environment [32]. A further functional analysis of these *P. monticola* novel defence-related genes would provide novel insight into resistance mechanisms of this conifer.

### ABA signalling involved in the *Cr2*-mediated resistance

Among other intermediate factors potentially functioning in *Cr2*-triggerred signalling, we detected significant up-regulation of transcripts for ABA receptor, ABA 8-hydroxylase, GRAM-containing ABA-responsive protein, and annexin proteins in resistant seedlings post *C. ribicola* infection (Additional file 5: Table S13). Apart from its central role in plant development, ABA plays a modulating role in diverse plant-pathogen interactions mediated at least in part by crosstalk with JA and SA [33]. ABA receptor proteins bind and respond to the hormone by activating the transcription of ABA-responsive genes involved in plant stress responses [34], including PR10 proteins [35].

The GRAM (for glucosyltransferases, Rab-like GTPase activators, and myotubularins) domain is ubiquitous in glucosyltransferases, myotubularins, and other membrane-associated proteins in eukaryotes [36]. The pepper GRAM domain–containing ABA-responsive protein, ABR1, negatively regulates ABA signaling by suppressing ABA biosynthesis, but promotes SA and ROS production, ultimately leading to cell death and disease resistance [37]. The mutant plants of an ABA 8′-hydroxylase gene (*cyp707a3-1*) accumulated a higher level of stress-induced ABA with exaggerated ABA-inducible gene expression. ABA treatment suppresses induction of systemic acquired resistance (SAR) by inhibiting the SA pathway [38]. As signaling components with phospholipid binding ability, some annexin proteins are implicated in responses to ABA, oxidative, saline, cold, and pathogenic stress [39,40]. Consistent with these studies, we observed a dramatic up-regulation of *P. monticola* transcripts for five proteins in resistant seedlings: ABA receptor pyl8, GRAM-containing ABA-responsive protein, ABA 8-hydroxylase, ABA-responsive protein, and annexin homolog (Additional file 5: Table S13), suggesting that ABA-signalling may play a part in the *Cr2*-mediated resistance.

### Auxin signalling involved in the *Cr2*-mediated resistance

It is notable that 25 auxin-related transcripts were positively regulated by *C. ribicola* infection (Additional file 5: Table S13), and 15 of them were up-regulated specifically in resistant seedlings or with significantly higher expression levels in resistant seedlings than in susceptible seedlings, including two TFs (ARF2 and IAA13). ARF2 belongs to a TF family that binds to auxin-responsive elements (AuxREs) in the promoters of auxin-regulated genes, and acts as the intermediate factor for crosstalk between the primary signaling pathways of auxin and ABA [41]. Auxin itself is known as a virulence factor to suppress host defense in some plant pathosystems [42,43]. Pathogen-secreted indole-3-acetic acid (IAA) increases rice disease symptoms by inducing expansins that cause loosening of the cell wall [44]. White pine bark tissues increase a few-fold in thickness as fusiform swelling in the cankered regions (especially at aeciopsore stage) in susceptible seedlings, suggesting that *C. ribicola* may release plant hormone(s) to induce proliferation of host bark cells in responsive (i.e. susceptible) hosts [8]. In *Cr2*-resistant seedlings we found dramatic up-regulation (the highest with >1,000 folds increase) of a number of genes that down-regulate auxin and cellulose synthase-like protein (Additional file 4: Table S5), but expansin genes were highly suppressed (>10-fold decrease, Additional file 4: Table S6), indicating that auxin may have complex roles in the WP-BR interaction, and suppression of auxin action may be another important mechanism underlying the *Cr2*-mediated resistance by suppressing the loosening of the pine cell wall.

### The ubiquitin–proteasome system (UPS) was regulated by *C. ribicola* infection

UPS is a protein degradation system worthy of special attention in the WPBR pathosystem because all 22 related DEGs were positively regulated by *C. ribicola* infection and 14 of them were up-regulated only in resistant seedlings (Additional file 5: Table S13). The UPS-related genes specifically regulated in the resistance response include various types of ubiquitin-conjugating enzymes and ubiquitin-associated (UBA) zinc-finger (ZZ) and phox/Bem1p (PB1) domain proteins. The UPS regulates proteins of the ABA receptor-signal complex and its downstream targets. Most F-box proteins (FBPs) are characterized as components of the SCF (SKp1, Cullin, F-box protein) E3 ubiquitin–ligase complex, which participates in the recognition and recruitment of target proteins for ubiquitination and degradation by the 26S UPS. Expression of many FBPs is transcriptionally regulated in a temporal and tissue specific manner, or in response to (a)biotic stressors [45]. Auxin binding to the SCF complex results in enhanced removal of TF repressors belonging to the AUX/IAA family through the SCF E3-ubiquitin ligase proteasome (26S) pathway [46]. The degradation of the AUX/IAA transcriptional repressors leads to the activation of ARFs and the expression of auxin-responsive genes, which, in turn, positively regulate plant resistance to necrotrophic fungi in Arabidopsis [47]. The proteins of the plant p62/NBR1/Joka2 family contain PB1, ZZ and C-terminal UBA domains and presumably function as cargo receptors in the selectivity of autophagy, which may constitute an important part of plant response to environmental stresses [48]. Accumulated evidence has identified subunits and proposed regulators of SCF ubiquitin ligases as essential components of R gene-mediated resistance [49]. Up-regulation of the UPS- and FBP-related transcripts may help white pine to reprogram cellular homeostasis by recovering normal conformation of proteins and enzymes for resistance to *C. ribicola* invasion.

### Differential regulation of multiple TF families in the WPBR pathosystem

Down-stream defense-responsive genes are normally regulated positively or negatively by different TFs that are direct or indirect targets of various signal transduction pathways. The *P. monticola* TFs differentially expressed between compatible and incompatible interactions belong to a few superfamilies with well-characterized structural domains such as ARF2, IAA13, AP2, zinc-finger CCCH type, zinc-finger-HD, PLATZ, NAC, DOF zinc-finger, AP2/ERF-B3, R2R3-MYB, WRKY, C3HL, HD-leucine-zipper, and tubby-like F-box. Other genes potentially involved in regulating transcription processes include nuclear TF Y subunit b-3, cold-shock DNA-binding protein, ethylene-responsive transcriptional co-activator, and other DNA-binding proteins (Additional file 5: Table S13).

In addition to ARF2 and IAA13 functioning in auxin-signalling, we found five other families of TFs (zinc finger HD, NAC, PLATZ-domain, Dof-zinc finger, and tubby-like F-box) responsive specifically in *Cr2*-resistance. A soybean zinc finger homeodomain TF, GmZF-HD1, activates the expression of the calmodulin GmCaM4 in response to pathogens by specifically interacting with A/T-rich repeats in the promoter [50]. PLATZ is a class of plant-specific zinc-dependent DNA-binding protein responsible for A/T-rich sequence-mediated transcriptional repression [51]. The Dof TF family is involved in the control of a variety of plant-specific processes, including defense response, phytochrome signaling, and phenylpropanoid metabolism in an environmental and tissue-specific manner [52]. Six rice NAC genes showed preferential expression under biotic stress [53]. Arabidopsis NAC TFs may play a dual role in regulating both JA- and ABA-dependent responses [54]. An Arabidopsis NAC TF (ATAF1) functions as an attenuator of ABA signalling for the mediation of efficient penetration resistance upon *Blumeria graminis* attack [55]. NAC TFs manipulate plant stress responses by activating other genes encoding R2R3-MYB TF, amylase, cold responsive protein, dehydration responsive proteins, GST, and late embryogenesis abundant (LEA) proteins [56]. We observed significant regulation of two NAC homologs as well as GST, R2R3-MYB, and LEA genes following *C. ribicola* infection. Whether the latter are the targets of the *P. monticola* NAC TFs is an interesting question to address in future work.

*P. monticola* TF genes of six families (TF 3, C3HL domain class, AP2/ERF-B3 domain-containing, R2R3-MYB, CCCH type zinc finger, and HD-leucine zipper) were up-regulated in both resistant and susceptible seedlings. Two DEGs were detected in each family of the TFs with AP2/ERF-B3, R2R3-MYB, or CCCH type zinc finger domain, respectively. One family member was responsive in both resistant and susceptible seedlings while another was responsive only in resistant seedlings. Plant zinc-finger CCCH type TFs have been implicated to function in a series of plant developmental and adaptive processes, including plant defense responses to (a)biotic stresses, by regulating gene expression from the transcriptional to posttranscriptional levels [57,58]. A loss-of-function mutant of an Arabidopsis zinc finger CCCH domain-containing protein showed an increased local susceptibility to a fungal pathogen and sensitivity to seed germination in the presence of ABA [57]. Arabidopsis R2R3-MYB TF (MYB12) directly acts on the promoters of the flavonoid biosynthesis genes and it is placed at the downstream end of the signalling chain that causes flavonol-specific gene activation in phenylpropanoid biosynthesis [59]. Wheat R2R3 MYB TF (TaPIMP1) mediates host resistance to fungal pathogen and drought stresses by regulating defense-related genes (PR1 and PR5 genes) through ABA- and SA-signalling pathways [60]. Accompanying differential expression of *P. monticola* R2R3-MYB genes, we also observed differential expression of genes encoding enzymes related to biosynthesis of flavonol and phenylpropanoid, as well as genes encoding for multiple members of cytochrome p450, the TLP (PR5), PR10, and other PR families between resistant and susceptible genotypes.

Plant TFs with one AP2/ERF domain together with one B3 domain are assigned to the RAV (related to ABI3/VP1) family belonging to the plant AP2/ERF superfamily, and RAV family TFs have been reported to respond to hormones (ethylene and brassinosteroid) and (a)biotic stresses [61,62]. Over-expression of a RAV gene induced expression of ERF (ethylene-responsive factor) and PR5 genes and increased tolerance to bacterial pathogen in transgenic tomato [62]. *P. monticola* ACC oxidase genes were up-regulated and one ethylene-responsive transcriptional coactivitor was down-regulated in similar patterns in both resistant and susceptible seedlings, suggesting ethylene-signalling may be involved only in the basal response in the WPBR pathosystem. However, two *P. monticola* RAV (AP2/ERF-B3 domain) homologs showed differential expression between *Cr2/-* and *cr2/cr2* genotypes, suggesting their up-stream intermediate factors (such as MAPK and CIPK as candidates) may be different between two genotypes.

## Conclusions

In the present study WWP primary needles pooled from multiple seedlings at early stages (0- and 4-dpi) post *C. ribicola* infection were used for cDNA library construction. Secondary needle, stem (bark), and other tissues from more mature WWP trees or from tissues at relatively late pathogen infection stages could be investigated to detect tissue-specific defence responses and to identify host genes regulated specifically during other infection phases of the *C. ribicola* life cycle. These host genes may be found to contribute more to quantitative disease resistance. In this study, about 2.5% of the whole transcriptome assembly was identified as rust-responsive genes and 85% of them were functionally annotated in *P. monticola* defense, but their putative contribution to host resistance to *C. ribicola* awaits verification by functional genomics studies or association studies focused on exploring gene variation in *P. monticola* populations.

RNA-seq analysis of the WP-BR interactions revealed that (1) two types of plant R candidates (NBS-LRR and RLKs) were up-regulated specifically in resistant genotype following *C. ribicola* infection, suggesting a distinct role of these R candidates in *Cr2*-mediated resistance; (2) the biosynthesis and signalling pathways of multiple plant hormones (auxin, ABA, and ET) were coordinately regulated following rust infection, indicating that the auxin and ABA-mediated signaling pathways are involved in white pine resistance to rust; 3) a set of novel TFs were identified in response to *C. ribicola* infection, some of them (NAC, PLATZ, Dof, and ZF-HD TFs) specifically responsive in the incompatible WP-BR interaction; and 4) several families of PR proteins (PR1, PR2, PR3, PR5, PR6), ROS-related proteins (GSTs, thioredoxin-like and peroxidises), UPS proteins, and retrotransposons were differentially expressed at the transcriptional level between resistant and susceptible genotypes following *C. ribicola* infection.

## Methods

### Plant materials and rust inoculation

Inoculation of *P. monticola* seedlings with *C. ribicola* was the same as described previously [7]. In brief, 200 six-month-old seedlings of the open-pollinated seed lot #3926 (from a heterozygous resistant tree) were inoculated with *C. ribicola* in an inoculation chamber designed to facilitate basidiospore shed with day temperatures of 16°C and night temperatures of 12°C, respectively. Basidiospores were shed from *C. ribicola*-infected *Ribes nigrum* leaves that were laid on a metal mesh above the white pine seedlings. Infected leaves were collected from a *Ribes* garden on Vancouver Island where only pathogenic *avcr2* isolates were available. Basidiospore density was monitored by placing glass slides at random underneath the mesh during rust inoculation. WWP needles were inoculated at a spore density higher than 3,000 spores per square centimeter. Primary needles were collected from at least 10 seedlings individually for each treatment at 0- and 4-dpi and stored at -80°C. Each seedling was pre-identified as resistant (*Cr2/-*) or susceptible (*cr2/cr2*) genotype using *Cr2*-linked DNA markers as reported previously [63].

### cDNA library construction

Total RNA was extracted from approximately five grams of needles pooled from at least 10 seedlings per treatment using a protocol described previously [64]. After eliminating traces of genomic DNA by treatment with DNase I (Invitrogen, Burlington, ON, Canada), an automated capillary gel electrophoresis was used to assess RNA quality and quantity using a Bioanalyzer 2100 with RNA 6000 Nano Labchips (Agilent Technologies Ireland, Dublin, Ireland). Total RNA samples with 28S/18S ratios in a range from 1.8 to 2.0 and RNA integrity index from 8.0 to 10.0 were selected for further processing.

PolyA?+?RNA fraction was purified from total RNAs (~5 μg) using a MACS mRNA isolation kit (Miltenyi Biotec, Bergisch Gladbach, Germany). Double stranded cDNA was synthesized using a Superscript cDNA synthesis kit (Invitrogen) with random hexamer primers at a concentration of 5 μM. The cDNA fragments were sheared using a Covaris E110 (Covaris, Woburn, MA, USA) for 75 sec under conditions “Duty cycle” of 20% and “Intensity” of 5, then the cDNA fraction with lengths of 200-250 bp was excised from 8% polyacrylamide gel electrophoresis (PAGE) for cDNA library construction using a paired-end sample prep kit (Illumina, San Diego, CA, USA). Briefly, the cDNAs were subject to end-repair and phosphorylation by T4 DNA polymerase, Klenow DNA polymerase, and T4 polynucleotide kinase respectively in a single reaction. The cDNAs with 3′-A overhangs, generated by Klenow fragment (3′ to 5′ exo minus), were ligated to paired-end adapters, which contain 5′-T overhangs. The adapter-ligated products were purified and enriched by PCR for 10 to 15 cycles using Phusion DNA polymerase and paired-end primer set (Illumina). PCR product of the desired size range was purified using 8% PAGE. The purified cDNA quality was assessed and quantified using an Agilent DNA 1000 series II assay kit on the Agilent 2100 Bioanalyzer (Agilent) and a Quant-IT ds-DNA HS assay kit on the Qubit fluorometer (Invitrogen). The cDNA library was diluted to 8 nM and this final concentration was checked and determined again by Quant-IT dsDNA HS Assay before Illumina sequencing.

### Illumina RNA-seq analysis

The Illumina GA IIx platform was used for deep sequencing of the cDNA libraries from both 5′- and 3′- end for 76-bp reads following the manufacturer’s manual at the British Columbia Cancer Agency (Vancouver, BC, Canada). The deconvolution of fluorescent images to DNA sequences, base-calling and quality value calculation were performed using the Illumina data processing pipeline (version 1.4). The raw Illumina 76-bp pair-end sequences were deposited in the NCBI Sequence Read Archive (SRA) under accession numbers SRR1013833, SRR1013836, and SRR1013837.

### Bioinformatics analyses

The CLC genomics workbench (version 5.5; CLC bio, Aarhus, Denmark) was selected for *de novo* transcriptome assembly in the present study because the CLC software has a faster computing pace with comparable or better assembly results than other bioinformatics programs [65]. Reads and read stretches of poor quality bases were removed and trimmed with filter threshold at quality score (Q ≥0.05), ambiguous nucleotide (N ≤2), and length trimming (N ≥30) before *de novo* transcriptome assembly.

Using the BLASTn and tBLASTx algorithms, all non-redundant contigs were used for a BLAST search against the NCBI nr database (http://www.ncbi.nlm.nih.gov/), the PGI or SGI database (http://compbio.dfci.harvard.edu/tgi/tgipage.html) [66], a *Melampsora_laricis populina* protein database (http://genome.jgi-psf.org/Mellp1/Mellp1.download.ftp.html), and a set of *P. monticola* ESTs (Girard-Martel M, personal communication). The PGI (Release 9.0, March 26, 2011) and SGI databases (Release 5.0, March 30, 2011) contained 77,326 and 79,409 unique ESTs respectively. GO annotation assignment [67] was used to perform functional gene annotation by mapping GO terms using databases of the NCBI nr, PIR (http://pir.georgetown.edu/pirwww/), GO (http://www.geneontology.org/), UniProts (http://www.ebi.ac.uk/UniProt/), and KEGG (http://www.genome.jp/kegg/) in the BLAST2GO program (Biobam Bioinformatics S.L., Valencia, Spain, http://www.blast2go.com/b2ghome/about-blast2go) with an E-value cutoff of 10^-6^[68]. Biological pathways were identified by gene annotation using enzyme code and KEGG databases [69]. GOslims_Plant was used to generate a focused view of the plant GO categories (http://www.geneontology.org/GO_slims/). A consensus transcriptome was created by assembly comparison of three cDNA libraries constructed from uninfected WWP needles (as control at 0-dpi), *C. ribicola*-infected needles from resistant (*Cr2/-*) and susceptible (*cr2/cr2*) genotypes at 4-dpi following a procedure of reciprocal BLASTn as described by Ness et al. [70].

Raw reads data were mapped back to the reference transcriptome for evaluation of gene expression levels and global transcript expression profiling among plant samples with different treatments. For statistical analysis to identify DEGs, RPKM was calculated as the normalized transcript expression value [71]. To determine whether cDNA libraries were compatible with each other, a box plot and a density plot were performed to evaluate the RPKM overall distribution, variability, and similarity in each sample using Bioconductor (version 2.12) software in conjunction with R software (version 3.0.0). A Z-test [72] was used to identify DEGs between any two experimental conditions (at a Bonferroni corrected *p*-value cut-off of 0.05) using the CLC genomics workbench. The expression patterns of DEGs were analyzed by a K-means clustering method [73] using euclidean distance based on gene expression values over all input samples (Additional file 5: Table S14).

### Transcript expression analysis via qRT-PCR

A subset of contigs assembled from RNA-seq was used for transcript expression analysis via qRT-PCR. qRT-PCR analysis was performed as described previously [7,14,18]. Gene-specific primers of 26 genes were designed (Additional file 5: Table S15), including actin and tubulin genes as internal controls. Student t tests were used to analyze the significance of transcript differences between control and infected samples. Correlation/regression analyses were performed to compare fold changes of transcripts measured by qRT-PCR and RNA-seq analysis. ANOVA tests were used to estimate statistical significance of correlation between two sets of expression data generated by RNA-seq analysis and qRT-PCR.

## Competing interests

The authors declare that they have no competing interests.

## Authors’ contributions

JJL conceived the study, performed analysis of RNA-seq data, and drafted the manuscript. RB performed statistical analysis. RS conceived and helped to facilitate construction of the bioinformatics platform. All authors read and provided comments and approved the final manuscript.

## Supplementary Material

Additional file 1 **Table S1.** RNA-seq reads generated and analyzed in three pooled western white pine primary needles. **Table S2.** BLAST search to evaluate transcriptome data sets of western white pine primary needles. **Table S3.** Top ten sequences with most abundant expression levels based on total gene reads or their RPKM values. **Table S4.** Gene and enzyme numbers of metabolic pathways detected in the whole primary needle transcriptome and differentially expressed genes (DEGs) regulated in the white pine-blister rust (WP-BR) interactions. Click here for file

Additional file 2: Figure S1 Plot analysis of transcript expression values (RPKM) in three western white pine cDNA libraries for quality control. The RPKM overall distribution and variability of three cDNA libraries/samples were similar, indicating that they were comparable for identification of differentially expressed genes (DEGs) at the transcriptome level. (A) A box plot analysis using CLC genomics work bench; (B) A density plot using Bioconductor (version 2.12) software in conjunction with R software (version 3.0.0). Click here for file

Additional file 5 **Table S13.** Functional grouping of differentially expressed genes (DEGs) during early stages of compatible and incompatible white pine-blister rust (WP-BR) interactions. **Table S14.** A list of contigs used in the K-means clustering analysis. **Table S15.** A list of primers used for quantitative reverse transcriptase-polymerase chain reaction (qRT-PCR). Click here for file

Additional file 3: Figure S2 Functional classification of the differentially expressed genes (DEGs) in white pine-blister rust (WP-BR) interactions at an early stage (4-dpi) post *Cronartium ribicola* inoculation. Subcategories (A) for biological process (BP), (B) for molecular function (MF), and (C) for cellular component (CC). Click here for file

Additional file 4 **Table S5.** A list of 245 contigs with significant up-regulation specifically in resistant (*Cr2/ -)* seedling post *C. ribicola* infection (4-dpi). **Table S6.** A list of 142 contigs with significant down-regulation specifically in resistant (*Cr2/ -)* seedling post *C. ribicola* infection (4-dpi). **Table S7.** A list of 114 contigs with significant up-regulation specifically in susceptible (*cr2/cr2*) seedling post *C. ribicola* infection (4-dpi). **Table S8.** A list of 46 contigs with significant down-regulation specifically in susceptible (*cr2/cr2*) seedling post *C. ribicola* infection (4-dpi). **Table S9.** A list of 204 contigs with significant up-regulation in both resistant (Cr2/ -) and susceptible (*cr2/cr2*) seedling post *C. ribicola* infection (4-dpi). **Table S10.** A list of 106 contigs with significant down-regulation in both resistant (*Cr2/ -*) and susceptible (*cr2/cr2*) seedling post *C. ribicola* infection (4-dpi). **Table S11.** A list of 141 contigs with significantly higher expression levels in resistant (*Cr2/ -)* seedlings than in susceptible (*cr2/cr2*) seedling post *C. ribicola* infection (4-dpi). **Table S12.** A list of 134 contigs with significantly higher expression levels in susceptible (*cr2/cr2*) seedling than in resistant (*Cr2/ -*) seedlings post *C. ribicola* infection (4-dpi). Click here for file
