# Supplementary figures and images for: Transcriptome analysis of Pinus monticola primary needles by RNA-seq provides novel insight into host resistance to Cronartium ribicola
Source: BMC Genomics. 2013 Dec 16;14:884. doi: 10.1186/1471-2164-14-884 (PMC3907366; doi:10.1186/1471-2164-14-884)

## Slide 1
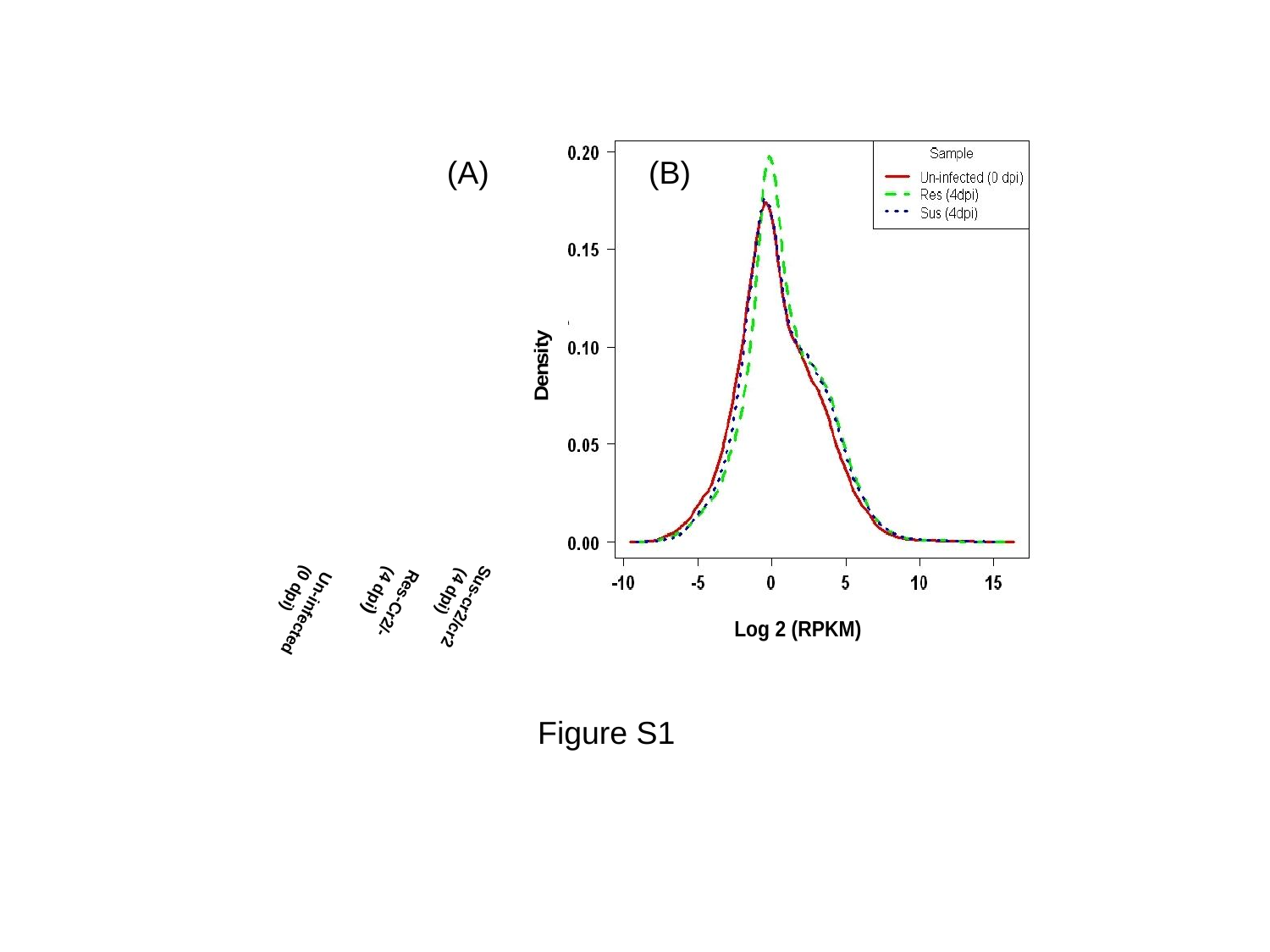

(A) (B)
Figure S1

Supplement: Additional file 2: Figure S1 — Plot analysis of transcript expression values (RPKM) in three western white pine cDNA libraries for quality control. The RPKM overall distribution and variability of three cDNA libraries/samples were similar, indicating that they were comparable for identification of differentially expressed genes (DEGs) at the transcriptome level. (A) A box plot analysis using CLC genomics work bench; (B) A density plot using Bioconductor (version 2.12) software in conjunction with R software (version 3.0.0). [file 1471-2164-14-884-S2.ppt]

## Slide 1
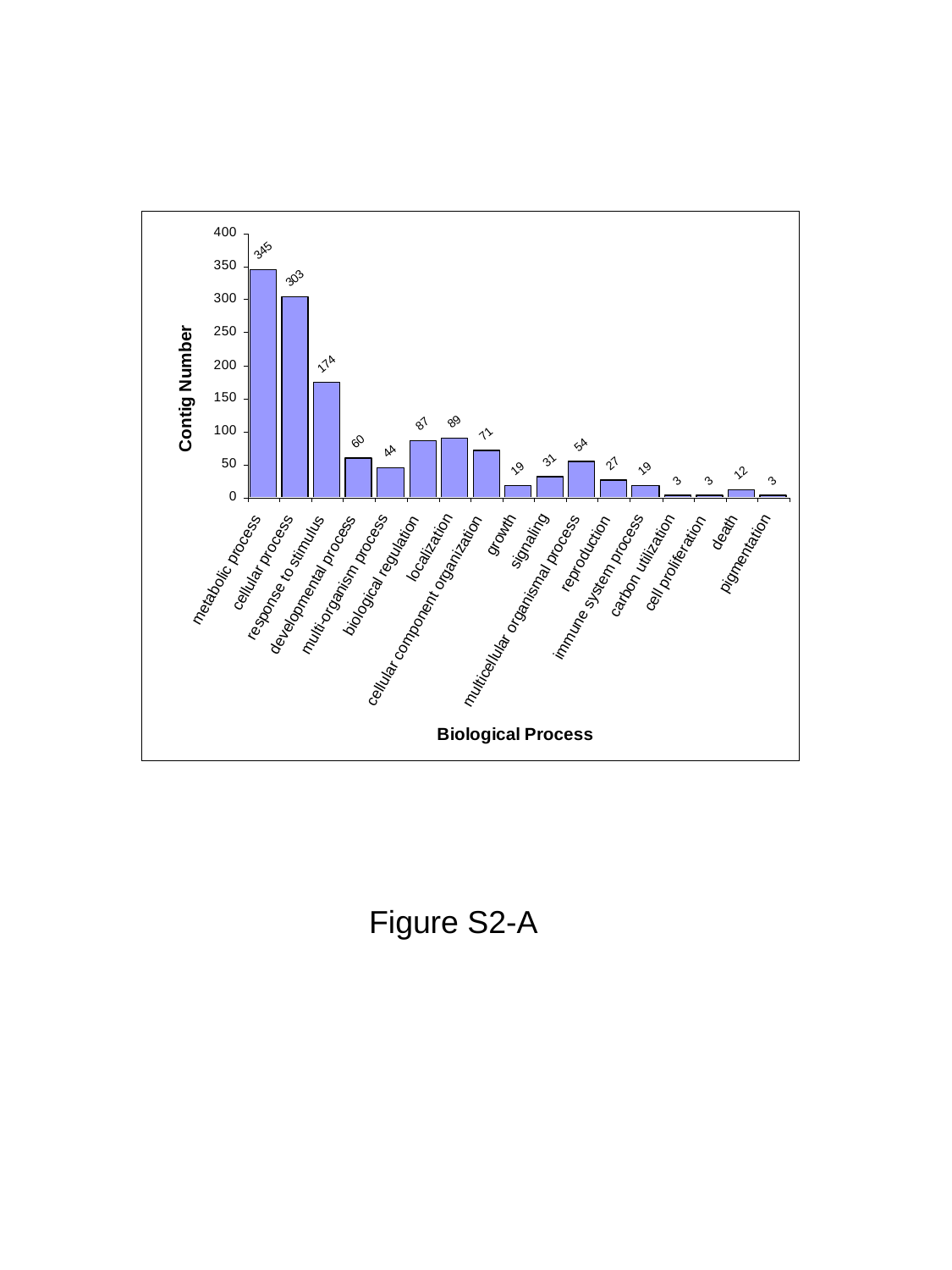

Figure S2-A

## Slide 2
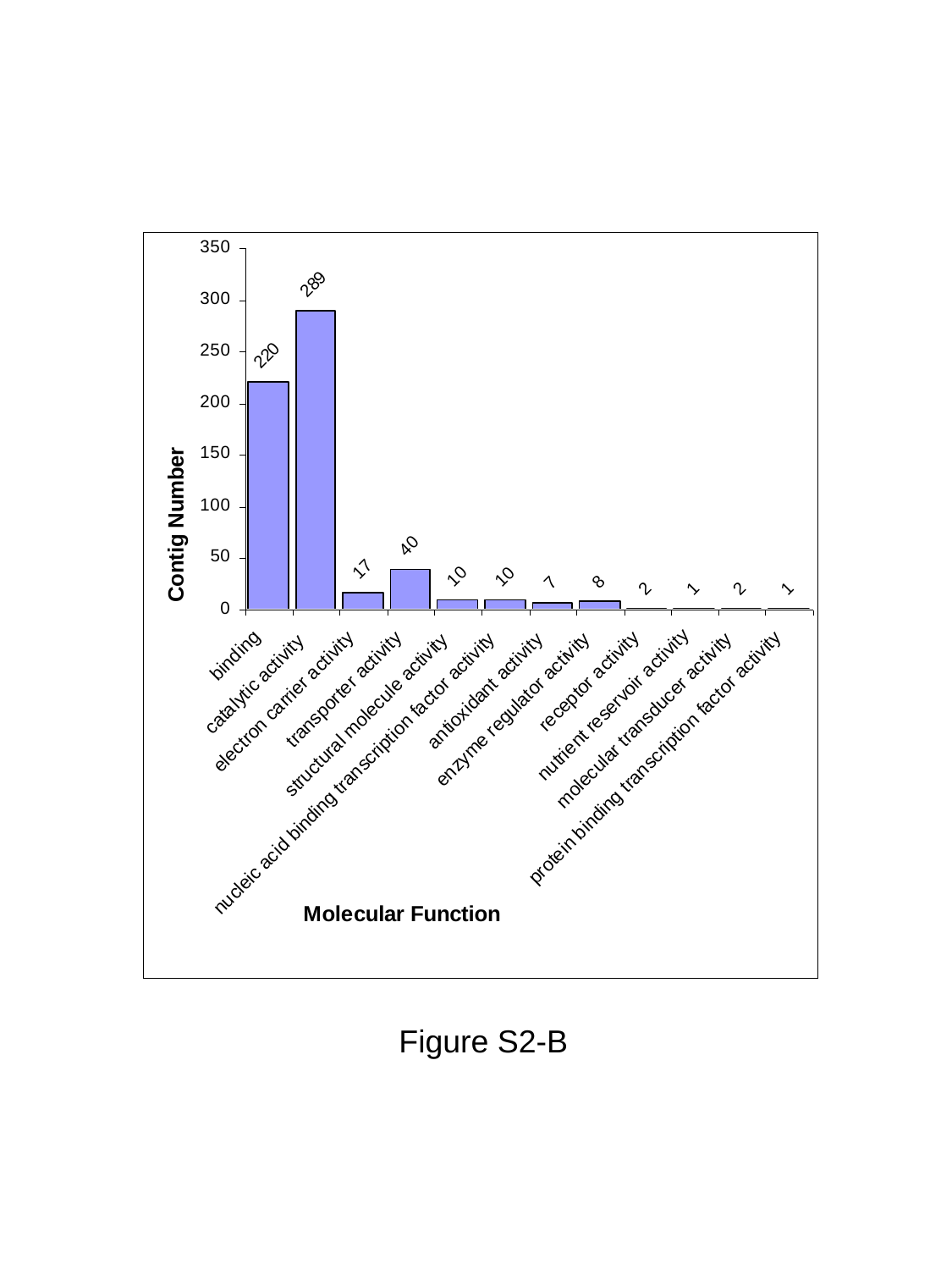

Figure S2-B

## Slide 3
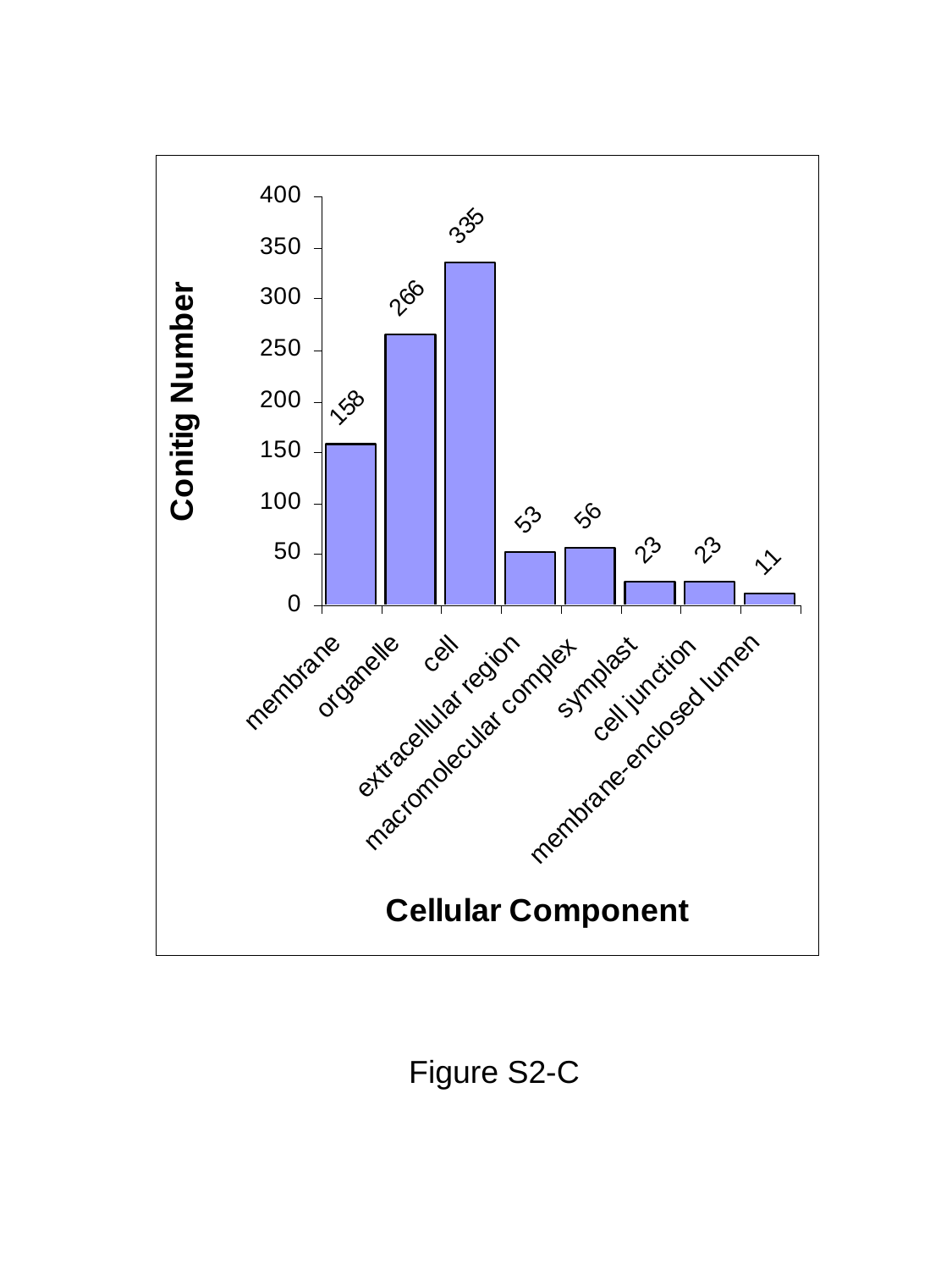

Figure S2-C

Supplement: Additional file 3: Figure S2 — Functional classification of the differentially expressed genes (DEGs) in white pine-blister rust (WP-BR) interactions at an early stage (4-dpi) post Cronartium ribicola inoculation. Subcategories (A) for biological process (BP), (B) for molecular function (MF), and (C) for cellular component (CC). [file 1471-2164-14-884-S3.ppt]
